# Small RNA-Seq to Characterize Viruses Responsible of Lettuce Big Vein Disease in Spain

**DOI:** 10.3389/fmicb.2018.03188

**Published:** 2018-12-21

**Authors:** Agustina Bernal-Vicente, Livia Donaire, Covadonga Torre, Cristina Gómez-Aix, Maria Amelia Sánchez-Pina, Miguel Juarez, Yolanda Hernando, Miguel A. Aranda

**Affiliations:** ^1^Department of Research and Development, ABIOPEP S.L., Murcia, Spain; ^2^Biology of Stress and Plant Pathology Department, CEBAS-CSIC, Murcia, Spain; ^3^Plant Production and Microbiology Department, University Miguel Hernández of Elche, Orihuela, Spain

**Keywords:** lettuce, LBVaV, MiLBVV, big-vein disease, small RNA-seq, qRT-PCR

## Abstract

The emerging lettuce big-vein disease (LBVD) is causing losses in lettuce production ranging from 30 to 70% worldwide. Several studies have associated this disease with Mirafiori lettuce big-vein virus (MiLBVV) alone or in mixed infection with lettuce big-vein associated virus (LBVaV). We used Illumina small RNA sequencing (sRNA-seq) to identify viruses present in symptomatic lettuce plants from commercial fields in Southern Spain. Data analysis using the VirusDetect tool showed the consistent presence of MiLBVV and LBVaV in diseased plants. Populations of MiLBVV and LBVaV viral small RNAs (sRNAs) were characterized, showing features essentially similar to those of other viruses, with the peculiarity of an uneven asymmetric distribution of MiLBVV virus-derived small RNAs (vsRNAs) for the different polarities of genomic RNA4 vs. RNAs1 to 3. Sanger sequencing of coat protein genes was used to study MiLBVV and LBVaV phylogenetic relationships and population genetics. The Spanish MiLBVV population was composed of isolates from three well-differentiated lineages and reflected almost all of the diversity reported for the MiLBVV species, whereas the LBVaV population showed very little genetic differentiation at the regional scale but lineage differentiation at a global geographical scale. Universal primers were used to detect and quantify the accumulation of MiLBVV and LBVaV in field samples; both symptomatic and asymptomatic plants from affected fields carried equal viral loads, with LBVaV accumulating at higher levels than MiLBVV.

## Introduction

Lettuce big-vein is a damaging disease responsible for important quality and yield losses worldwide. Affected lettuce plants show chlorophyll clearing along the veins, causing a characteristic big-vein appearance, and also crinkled leaves, head size reduction and significant reduction of the quality of the edible product (Maccarone, 2013). Lettuce is increasingly cultivated all over the world due to, among other factors, the demand for fresh and healthy vegetable products, the diversification of varietal types and the expansion of the commercialization of ready-to-eat products. Lettuce big-vein disease (LBVD) can cause production losses of up to 70% in certain regions, and incidences close to 100% are frequent during the winter and early spring in specific areas of cultivation (Moreno and Fereres, 2012; Maccarone, 2013; our unpublished observations). The disease was first reported in California (USA) a long time ago (Jagger and Chandler, 1934), but today the biology of the microorganisms responsible for the disease is still not fully understood. For a long time, it was thought that lettuce big-vein associated virus (LBVaV; species *Lettuce big-vein associated virus*, genus *Varicosavirus*) was the LBVD causal agent (Kuwata et al., 1983). LBVaV is a bipartite, negative-sense, single-stranded (ss) RNA virus. The viral genome has seven open reading frames (ORFs) distributed into two ssRNAs, with a coat protein (CP) of 48 kDa encoded by RNA2 (Sasaya et al., 2002, 2004; King et al., 2012). Later, Roggero et al. (2000) isolated a filamentous virus, Mirafiori lettuce big-vein virus (MiLBVV; species *Mirafiori lettuce big-vein virus*, genus *Ophiovirus*) from symptomatic lettuce plants and proposed that MiLBVV, but not LBVaV, was the causal agent of LBVD, as plants infected with LBVaV did not develop symptoms in the absence of MiLBVV, whereas plants infected with MiLBVV developed big-vein symptoms regardless of the presence or absence of LBVaV (Lot et al., 2002; Sasaya et al., 2008). MiLBVV is a segmented negative-stranded RNA virus. The viral genome consists of four ssRNAs containing seven ORFs. RNA3 encodes a 48.5 kDa protein, which is the CP and a major component of its thin and filamentous particles (King et al., 2012). However, relatively recent data in the literature still questioned the LBVD etiology, as plants diagnosed positive for LBVaV but negative for MiLBVV by ELISA were symptomatic at least in a field experiment in Italy (Roggero et al., 2003); differential sensitivity of the detection method used for each virus may underlie this conflicting observation. Both viruses are transmitted by zoospores of a chytridiomycete fungus, *Olpidium virulentus*, a soil-borne obligate root parasite (Hartwright et al., 2010; Maccarone et al., 2010a). Resting spores can remain dormant in the soil and MiLBVV or LBVaV can survive up to 20 years inside them (Campbell, 1985). For this reason, inoculum of both viruses may last for years making LBVD control a challenge once the fungus is established in the field. Control strategies are also difficult, as *O. virulentus* infects a wide range of weed species which can act as reservoirs (Campbell and Fry, 1966; Navarro et al., 2005). Maccarone (2009) has detected the presence of MiLBVV and LBVaV in extracts of seeds collected from plants infected by both viruses, so seed transmission of these viruses is possible (Maccarone, 2009).

Spain is the fourth-largest lettuce producer in the world after China, the USA and India, and it is the number one exporter worldwide^1^. The area of lettuce cultivation in Spain is around 34,500 ha, with 15,630 ha (45.3% of the total) located in the Region of Murcia (Southeast Spain), followed by Almeria (20.8%) and Alicante (3.3%), with Murcia being the producer of ~70% of the Spanish exported lettuce^2^. MiLBVV and LBVaV detection has not been performed for the most comprehensive survey carried out in lettuce crops in Spain (Moreno et al., 2004), but the high proportion of negative detections for the viruses tested on samples from symptomatic plants in that survey, together with additional evidence (Navarro et al., 2004, 2005) suggest that the incidence of LBVD viruses in Spanish crops is high. ELISA and RNA hybridization-based methods have been set up for the detection of both viruses (Roggero et al., 2003; Navarro et al., 2004; Sasaya et al., 2008), as well as primers and probes for virus detection by reverse transcription-real time quantitative PCR (qRT-PCR) (Momonoi et al., 2015). Previously, the genetic variability of both viruses in Spain was studied based on the sequences of the CP genes of 7 and 11 MiLBVV and LBVaV Spanish isolates, respectively, in comparison with other isolates from around the world, suggesting more diverse populations for MiLBVV than for LBVaV (Navarro et al., 2005). The aim of our study was to use deep sequencing of small RNAs (sRNA-seq), a non-biased method, for identifying viruses infecting field plants affected by LBVD, and with this information, to develop techniques for the detection and characterization of the viruses present in field lettuce plants affected with LBVD and in commercial seed lots. We have also used the genetic information gathered to revisit the genetic variability and evolution of Spanish MiLBVV and LBVaV populations.

## Materials and Methods

### Plant Material and RNA Extraction

Lettuce crops in open fields near Águilas (Murcia, Southeast Spain) were surveyed during 2016, 2017, and 2018. For sRNA-seq and CP ORFs cloning and sequencing, three lettuce plants with LBVD-like symptoms were randomly taken from 4 different plots, and leaves and roots from these plants were sampled (Table 1). Plots ranged in size approximately between 2 and 6 ha; a single cultivar was predominantly planted in each plot and thus samples from each plot are from distinct cultivars: cv. Chavela (Enza Zaden) for plot “La Perla” (plot 1), cv. Zoliva (Nunhems) for plot “JB3” (plot 2), cv. Juanola (Enza Zaden) for plot “Primicias” (plot 3), and cv. Fernandola (Enza Zaden) for plot “La Serreta” (plot 4). Total RNA from roots and leaves was purified using TRI Reagent following the manufacturer's instructions (Sigma-Aldrich, USA). The concentration of total RNA was determined using a NanoDrop spectrophotometer (Thermo Fisher Scientific, USA) and its quality was checked with agarose gel electrophoresis.

**Table 1:** Samples used for sequencing in this study and cDNA clones obtained from these samples.

**Samples^a^**	**Analysis in which these samples have been used**	**cDNA clones for MiLBVV from samples**	**cDNA clones for LBVaV from samples**	**cDNA clones for MiLBVV from pool^b^**	**cDNA clones for LBVaV from pool^b^**
16_AG_1_L_1	RNA-seq, pool 1^b^ Phylogenetic analysis			Pool 1_M.1 Pool 1_M.2 Pool 1_M.3 Pool 1_M.4	Pool 1_L.1 Pool 1_L.2 Pool 1_L.3 Pool 1_L.4
16_AG_1_L_2				
16_AG_1_R_2				
16_AG_1_R_3				
16_AG_2_L_1	RNA-seq, pool 2^b^ Phylogenetic analysis			Pool 2_M.1 Pool 2_M.2 Pool 2_M.3 Pool 2_M.4	Pool 2_L.1 Pool 2v_L.2^g^ Pool 2_L.3 Pool 2_L.4^g^
16_AG_2_R_1			16_AG_2_R_1_L.1		
16_AG_2_R_2					
16_AG_2_L_3					
16_AG_2_R_3		16_AG_2_R_3_M.1 16_AG_2_R_3_M.2 16_AG_2_R_3_M.3 16_AG_2_R_3_M.4	16_AG_2_R_3_L.1^c^ 16_AG_2_R_3_L.2^c^ 16_AG_2_R_3_L.3 16_AG_2_R_3_L.4^c^		
16_AG_3_R_1	RNA-seq, pool 3^b^ Phylogenetic analysis			Pool 3_M.1 Pool 3_M.2 Pool 3_M.3 Pool 3_M.4 Pool3_M.5^i^ Pool3_M.6^i^ Pool3_M.7^i^ Pool3_M.8^i^	Pool 3_L.1^h^ Pool 3_L.2 Pool 3_L.3 Pool 3_L.4^h^	
16_AG_3_L_2			16_AG_3_L_2_L.1 16_AG_3_L_2_L.2 16_AG_3_L_2_L.3 16_AG_3_L_2_L.4		
16_AG_3_R_2					
16_AG_3_L_3				
17_AG_4_L_1	Phylogenetic analysis	17_AG_4_L_1_M.1 17_AG_4_L_1_M.2 17_AG_4_L_1_M.3 17_AG_4_L_1_M.4	17_AG_4_L_1_L.1^d^ 17_AG_4_L_1_L.2^d^ 17_AG_4_L_1_L.3 17_AG_4_L_1_L.4^d^		
18_AG_4_L_1	Phylogenetic analysis	18_AG_4_L_1_M.1 18_AG_4_L_1_M.2 18_AG_4_L_1_M.3	18_AG_4_L_1_L.1 18_AG_4_L_1_L.2 18_AG_4_L_1_L.3 18_AG_4_L_1_L.4		
17_AG_Ø_S1_Ø	Detection Phylogenetic analysis		17_AG_Ø_S1_Ø_L.1 17_AG_Ø_S1_Ø_L.2^e^ 17_AG_Ø_S1_Ø_L.3^e^		
17_AG_Ø_S2_Ø	Detection Phylogenetic analysis	17_AG_Ø_S2_Ø_M.1 17_AG_Ø_S2_Ø_M.2	17_AG_Ø_S2_Ø_L.1 17_AG_Ø_S2_Ø_L.2 17_AG_Ø_S2_Ø_L.3 17_AG_Ø_S2_Ø_L.4		
17_AG_Ø_S3_Ø	Detection Phylogenetic analysis		17_AG_Ø_S3_Ø_L.1 17_AG_Ø_S3_Ø_L.2 17_AG_Ø_S3_Ø_L.3 17_AG_Ø_S3_Ø_L.4		
17_AG_Ø_S4_Ø	Detection Phylogenetic analysis		17_AG_Ø_S4_Ø_L.1 17_AG_Ø_S4_Ø_L.2 17_AG_Ø_S4_Ø_L.3^f^ 17_AG_Ø_S4_Ø_L.4^f^		
17_AG_Ø_S5_Ø	Detection				
17_AG_Ø_S6_Ø	Detection				
17_AG_Ø_S7_Ø	Detection				
17_AG_Ø_S8_Ø	Detection				
17_AG_Ø_S9_Ø	Detection				
17_AG_Ø_S10_Ø	Detection				
17_AG_Ø_S11_Ø	Detection				

a *The notation of each sample refers to: Year_location_plot_tissue_plant. The symbol Ø indicates absence of data*.

b *RNAs were extracted from individual plants and their quality and integrity checked independently; each pool was formed by four or five RNA extracts of RIN ≥8 from samples from leaves or roots from a plant of the same plot*.

c-i *Identical numbers denote identical nucleotide sequences. Only one sequence from each group of identical sequences has been used for phylogenetic analysis*.

For the detection and analysis of distribution of MiLBVV and LBVaV in leaves and roots, 26 lettuce plants from additional plots in Águilas were sampled during winter 2017. Of the 26 iceberg lettuce plants sampled, 14 showed no symptoms, even though LBVD-like symptoms were highly prevalent (~50%) in the surveyed plots. One hundred mg of tissue were taken from roots, as well as from young and old leaves from each lettuce plant sampled. Additionally, seeds from 11 different lettuce varieties from 5 commercial brands were analyzed. For each of the varieties, two samples of 30 seeds each were used. Seed pellets were taken out before RNA extraction because our preliminary tests showed interference from the compounds used in pelleting with nucleic acid extraction (data not shown). Total RNA for virus detection from lettuce leaves, roots and seeds was extracted using Nucleospin RNA plant Kit following the manufacturer's instructions (Macherey-Nagel, Germany).

### sRNA Library Construction and Bioinformatic Analyses

RNA integrity numbers (RIN) were determined for each RNA extract using a 2100 Bioanalyzer System (Agilent, USA); these figures were used to decide whether or not RNA samples could be used for sRNA-seq. Only RNA extracts of RIN ≥8 were mixed in pools; thus pools 1–3 (Table 1) are composed of 4–5 RNA extracts from leaves or roots from 3 different plants from plots 1–3 (see above), respectively. The RNA pools were sent to Fasteris Life Sciences (Switzerland) for sRNA-seq. sRNAs 18–30 nt in length were size-selected from 2 to 3 μg of total RNA from each pool by polyacrylamide gel electrophoresis and sRNA libraries were prepared using TruSeq Small RNA Library Prep Kit (Illumina). Equimolar amounts of each sRNA library were pooled for multiplexed sequencing in a single lane on an Illumina HiSeq 4000 instrument (50 bp, single-end). After adapter removal, the quality of the reads was assessed using FastQC^3^. Reads were filtered by length (16–31 nts) and non-coding RNAs from the Rfam database were removed using Bowtie (Langmead et al., 2009). To search for virus sequences in the datasets, sequenced reads from each pool were submitted to the VirusDetect v1.7 online tool (Zheng et al., 2017). The lettuce genome (UC Davis Genome Center, CA, USA) was used as the reference to subtract host sRNAs. The percentage of identity of the VirusDetect viral contigs against different MiLBVV and LBVaV CP reference sequences was calculated by BLASTN using a command line standalone BLAST+ program. Consensus sequences of CP genes of either MiLBVV and LBVaV were built using SAMtools mpileup and BCFtools (Sanger Institute) from BAM files determined by mapping the sRNA reads once again against the reference viral sequences using BWA aln algorithm (Li and Durbin, 2009). Raw sRNA-seq data have been deposited in the Sequence Read Archive of NCBI under identifier SRP169058.

To analyse virus-derived small RNAs (vsRNA) populations, sRNA reads were again mapped to each viral genome of the MiLBVV isolate LS301-S and the LBVaV isolate LS302 using Bowtie allowing no mismatches to retain only true vsRNAs. Counting of total and unique vsRNAs as well as size and orientation distributions and composition of the 5′ end nucleotide of vsRNAs were calculated using our own Perl Scripts^4^. Distribution plots of vsRNAs against viral genomes were displayed using MISIS (Seguin et al., 2014).

### Cloning and Sequencing of the CP ORFs

RNA samples for cloning came from the pools 1, 2, and 3 used for sRNA-seq, and also from individual samples included in the pools, two samples from plot 4 (see above), as well as different seed samples (Table 1). RT and PCR amplification of the complete CP ORFs were performed in a two-step reaction. First-strand cDNA was synthesized using reverse primers MiLBVV_CP_R and LBVaV_CP_R (Table 2), respectively, and Expand Reverse Transcriptase (Roche Applied Science, Germany) or SuperScript® IV Reverse Transcriptase (Invitrogen, USA) following the manufacturer's instructions. PCR was performed in a 50 μl reaction using primer pairs MiLBVV_CP_F/MiLBVV_CP_R and LBVaV_CP_F/LBVaV_CP_R (Table 2), respectively, and the Expand High Fidelity PCR System (Roche Applied Science). PCR products were analyzed by agarose gel electrophoresis and DNAs of the expected sizes (Table 2) were recovered from gels using the GeneClean turbo kit (MP Biomedicals, Europe) according to manufacturer's recommendations. PCR products were ligated into pGEM®-T EasyVector (Promega Corp., USA), following manufacturer's instructions. *Escherichia coli* Stellar Competent Cells (Clontech, Takara Bio, Europe) were transformed following the supplier's protocol. Four recombinant clones were chosen for each PCR product. DNA plasmids were purified with GeneJET Plasmid Miniprep Kit (Thermo Fisher Scientific, USA) following the manufacturer's protocol. The presence and size of inserts was confirmed with a *Eco*R1 restriction digest of purified plasmid DNA. Sequencing of inserts in plasmids was done by Sanger sequencing (Stab Vida S.L., Portugal) using universal and internal primers (LBVaV_CP_F2, LBVaV_CP_R2, MiLBVV_CP_F2, MiLBVV_CP_R2) (Table 2).

**Table 2:** Primer and probe sequences.

**Name^a^**	**Sequence (5^**′**^-3^**′**^)**	**Product size (bp)**	**References**
**qRT-PCR**
LBVaV_F	TCAGTGACGTCGTGGAAATC	105	This study
LBVaV_P	[6-FAM]AAGACTGCCGGGAAAGAATCCTGG[BHQ-1]^b^	
LBVaV_R	CGTCGGACAGTACRGAAAGYT^c^	
MiLBVV-167F	AATTTCTYTWGGTCTCATGACAA^c^	72	Momonoi et al., 2015
MiLBVV-205T	[6-FAM]ACAGGCTTC TCTTC[MGB]^b^	
MiLBVV-238R	TTTGCAGATGCYACCATGG^c^	
VP 383_F (Nadh4)	AGCGTGCTAATCCCTATGTTCAT	363	Navarro et al., 2004
VP 389_R (Nadh4)	TCGGTGGTTCCTGTTTGGAA	
RWMV_F	GAAGGCTTACTGTTGTGAATGG	106	This study
RWMV_R	CTCTTCTGTCTGCTGGAACTAA	
RWMV_R_2	TGAAGGTATCGAGTTAAGTGTGAG	132
MNSV_F	GTATCAGGGCGCGTTTGATGA		Abiopep S.L
MNSV_R	GAATTGTCTCCAGTGCCTTACCA		Abiopep S.L
**Cloning**
MiLBVV_R PG	GCAGTCCTTGGCARATTYTTA^c^	312	This study
LBVaV_R PG	CCTTGAATGGATACTCGGTCTT	498	This study
LBVaV CP_F	ATGGCACACCCCAAATTGAAG	1,194	This study
LBVaV_CP_R	TCAYTCCTTCACTGGTGTCTCTCCCT^c^	
LBVaV_CP_R2	AAGTTCTGTCCGTAGTTGAG	785
LBVaV_CP_F2	GGTATGCTGATTTCTGTAAGACCG	701
MiLBVV_CP_F	ATGTCAGGAGTATACAARGT^c^	1,314	This study
MiLBVV_CP_R	TCA TTT CTT HCC RTA AGC TGT C^c^	
MiLBVV_CP_F2	GAGCACAACTTCATATTTGATGT	792
MiLBVV_CP_R2	AAGACTTGACTTGGAAACAAAGAAG	794

a *F, forward primer; R, reverse primer*.

b *Dual-labeled fluorescent probe*.

c *Degeneracies: R: A or G; Y: C or T; W: A or T; H: A or C or T*.

Nucleotide sequences are deposited in the NCBI database under accession numbers MH894447 to MH894472 (MiLBVV) and MH894473 to MH894508 (LVBaV).

### Population Diversity and Phylogenetic Analyses

The Molecular Evolutionary Genetics Analysis Version 7.0 (MEGA7) software (Kumar et al., 2016) was used to prepare multiple alignments of nucleotide sequences. Substitution models with the lowest BIC (Bayesian Information Criterion) scores were selected. Phylogenies were generated using the Maximum Likelihood method (Felsenstein, 1981), with 1000 bootstrap replicates. A Bayesian analysis was performed using the Beast v.1.8.4 program (Bouckaert et al., 2014). The substitution models chosen for that analysis were the same as those used for the maximum likelihood analysis. The resulting trees were constructed using treeAnnotator (available in the BEAST package) and edited using FigTree v1.4.3. The pairwise genetic distances between sequences were calculated using the MEGA7 software as well. Clustal X (2.1), a windows interface for Clustal W, was used to determine the percent nucleotide identity matrix (Thompson et al., 1997). The degree of functional restriction for the preservation of the LBVaV and MiLBVV CP coding regions was estimated from the ratio of nucleotide diversities at non-synonymous vs. synonymous positions (dN/dS); we used the Nei-Gojobori method (Nei and Gojobori, 1986) and the Kimura-2 parameters (Kimura, 1980) for MiLBVV and LBVaV, respectively, according to indications by the MEGA7 software.

### Virus Detection by Conventional and Quantitative RT-PCR

Whenever possible, primers described in the literature were used for virus detection and quantification (Navarro et al., 2004; Momonoi et al., 2015). Otherwise, primers were designed in conserved regions of the CP encoding genes using the Primer3 program (v.0.4.0) (Table 2). sRNA-seq results were validated by semi-quantitative RT-PCR. One hundred ng of the same RNA prep used for sRNA library construction were used for RT-PCR. Primers for MiLBVV and LBVaV are described below. For RWMV we tried semi-quantitative RT-PCR using primer pairs RWMV_F/RWMV_R and RWMV_F/RWMV_R_2 (Table 2) but without success. For MNSV we used MNSV_F/MNSV_R (Table 2). To determine primer efficiency in MiLBVV and LBVaV qRT-PCRs, a standard curve was elaborated using a transcribed RNA synthesized in our laboratory for each virus. To clone the cDNA of the transcript, additional primers MiLBVV-167F/MiLBVV_R PG and LBVaV_F/LBVaV_R PG (Table 2) were designed in the region of the CP genes to RT-PCR amplify fragments of 312 bp for MiLBVV and 498 bp for LBVaV. The obtained PCR products were electrophoresed in a 0.7% agarose gel, purified and cloned as described below. Once the inserts were cloned in pGEM-T Easy, the plasmids were linearized and transcribed with T7 RNA Polymerase (Promega), following the manufacturer's instructions. After transcription, treatment with DNase (Promega) and precipitation of RNA with sodium acetate were carried out to remove plasmid DNA. The transcribed RNAs were checked by agarose gel electrophoresis and quantified. The standard curve was made from 5 serial 1:10 dilutions of each of the synthesized transcripts. The qRT-PCR reactions were carried out in a StepOnePlus Real-Time PCR System (Applied Biosystems, USA) in a final volume of 10 μl with KAPA SYBR FAST One-Step qRT-PCR Master Mix (2X) Kit (Kapa Biosystems, USA) and the primers LBVaV_F/LBVaV_R and MiLBVV-167F/MiLBVV-238R (Table 2). The PCR conditions were: reverse transcription at 42°C for 5 min, denaturation at 95°C for 3 min, and 40 cycles of 3 s at 95°C followed by 20 s at 60°C. Dissociation curves were used to evaluate the generation of non-specific products. Analysis of copy number, linear regression and melting curve analysis were performed with the StepOnePlus version 2.3 software. The housekeeping gene used as control was the mitochondrial NADH dehydrogenase subunit 4 (*Nadh4)* coding gene from lettuce; for this, primers used were VP383_F and VP389_R (Table 2). For MiLBVV and LBVaV detection in seeds, we used an additional method based on TaqMan probes (Table 2). Differences in virus accumulation among samples were analyzed by one-way or two-way ANOVA using Statgraphics Plus 5.1 software.

## Results

### Sequencing of sRNAs From Symptomatic Lettuce Plants

**Figure 1:**
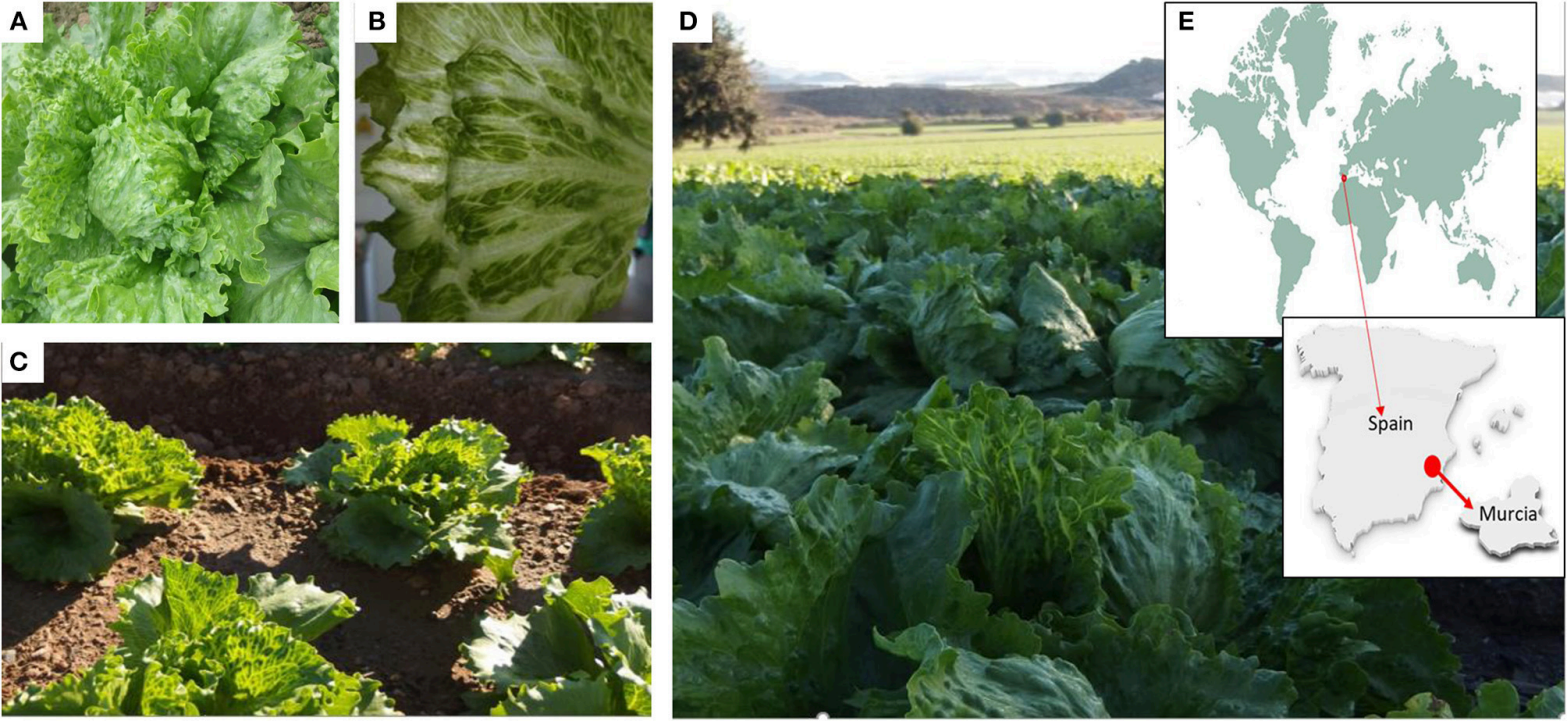
Symptomatic lettuce plants and crops, and location of the area surveyed. **(A,B)** Details of lettuce leaves with LBVD symptoms. Affected lettuce crops at the beginning **(C)** or at the end **(D)** of the cropping season. Surveys took place in Murcia **(E)** which is the main lettuce producing region in Spain.

**Figure 2:**
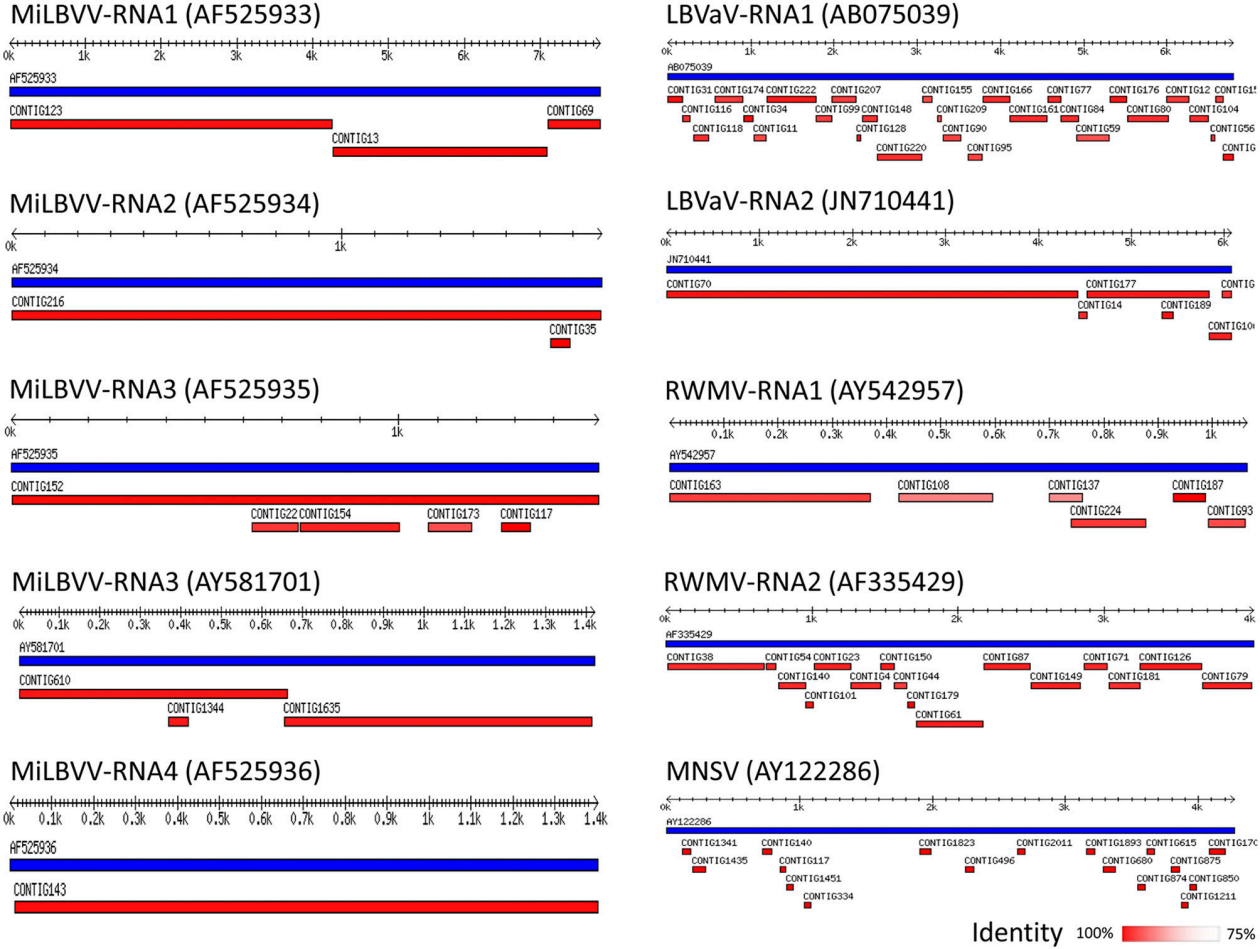
Schematic representation of virus contigs identified by VirusDetect. Distribution of contigs assembled by VirusDetect (in red) along the corresponding reference viral genomes (in blue). The accession numbers of the reference viral sequences are shown in brackets. The percentage of identity of the contigs with the reference sequences is shown as a color-scale. Tracks are those with better coverage, which corresponded to pool 2 except those matching accessions AY581701 and AY122286 that derived from pool 3.

We surveyed lettuce crops in open fields in Murcia (Southeast Spain), which is one of the major lettuce exporter regions in the world^1^. Surveys took place during 2016, 2017, and 2018, and our visual inspection suggested an incidence of LVBD-like symptoms close to 45% (Figure 1), with no obvious differences among lettuce cultivars. Three RNA pools from diseased plants from three different field plots (Table 1) were used for sRNA-seq, which produced 101–142 million reads (Table 3). The more abundant reads ranged in size from 18 to 26 nt, which fitted well with the canonical sRNA size classes. Reads of 16 to 31 nt were mapped against the Rfam database to discard non-coding RNA sequences. Thus, a total of 101,807,022; 79,763,445; and 71,875,996 reads in pools 1–3, respectively, were submitted to the VirusDetect online tool (Zheng et al., 2017) for virus identification using its plant virus database. From the total reads, 44–73% mapped to the lettuce genome, whereas 0.3–6% mapped to plant virus genomes (Table 3). Virus sRNA (vsRNA) reads could be assembled into 730, 226, and 2075 unique contigs in pools 1–3, respectively (Table 3). According to a VirusDetect BLASTN search, 101, 69 and 171 of those contigs could be mapped against 7, 10 and 8 different MiLBVV or LBVaV sequence accessions in each sample pool, respectively, with reference coverage ranging from 64.9 to 99.8% and average sRNA sequencing depth (normalized to reads per million) ranging from 0.4 to 66.9 (Supplementary Table 1 and Figure 2). In pools 1 and 2, contigs mapping to a second MiLBVV-RNA3 sequence were found but with a genome coverage below 21.3% (Supplementary Table 1 and Figure 2). VirusDetect also identified 24 contigs mapping to the RNA1 (AF335429), RNA2 (AY542957), and an unknown segment (AF335430) of ranunculus white mottle virus (RWMV; species *Ranunculus white mottle virus*, genus *Ophiovirus*, family *Aspiviridae*) genome in pool 2, with a reference coverage of 79.7–97.7% and a normalized sequencing depth of 1–15.2 (Supplementary Table 1 and Figure 2). Also, in pool 3, 17 contigs mapped to melon necrotic spot virus (MNSV; species *Melon necrotic spot virus*, genus *Alphacarmovirus*, family *Tombusviridae*) genome, with a coverage of 26.1% and a normalized sequencing depth of 0.2 (Supplementary Table 1 and Figure 2). Semi-quantitative RT-PCR was used to validate the presence of the above viruses in RNA pools; MiLBVV; and LBVaV were readily detected in all three pools, while neither RWMV nor MNSV could be confirmed in spite of the use of two primer pairs for the former.

**Table 3:** Summary of VirusDetect results of the three sample pools.

**Samples**	**Adapter removing**	**Filtering by length (16–31 nts)**	**Rfam mappings**	**Submited to VirusDetect**	**Lettuce mappings**	**Virus mappings**	**Unique contigs**	**Unique contigs mapping to viruses**	**Viruses identified by BLASTN**
Pool 1	140,978,643	135,217,018 (95.91%)	33,409,996 (24.71%)	101,807,022	58,530,638 (57.49%)	322,041 (0.32%)	730	101	7
Pool 2	101,966,562	96,932,063 (95.06%)	17,168,618 (17.71%)	79,763,445	58,038,873 (72.76%)	4,582,701 (5.75%)	226	69	10
Pool 3	107,243,009	97,725,818 (91.13%)	25,849,822 (26.45%)	71,875,996	31,930,149 (44.42%)	206,774 (0.29%)	2,075	171	8

Since the plant-virus database used by VirusDetect was processed to remove redundant sequences, a new BLASTN search was conducted to identify sequences closer to the viral sequences identified in our samples. For this, the viral contigs retrieved by VirusDetect were mapped against the CP sequences of different isolates of MiLBVV (22 sequences) and LBVaV (30 sequences) collected from NCBI, and their percentages of identity were recorded (Supplementary Table 2). Coverage was 100% in all cases. The highest percentage of identity was found for contigs mapping the CP sequence of isolate MiLBVV-LS301-O for all pools (97–99.6%) (Supplementary Table 2). In pool 3, other assembled contigs shared the highest sequence identity (~98.3%) to four isolates from the South of Spain (Supplementary Table 2; Navarro et al., 2005), suggesting the presence of isolates of different genetic groups in this pool.

### Characterization of Viral Small RNA Populations

**Figure 3:**
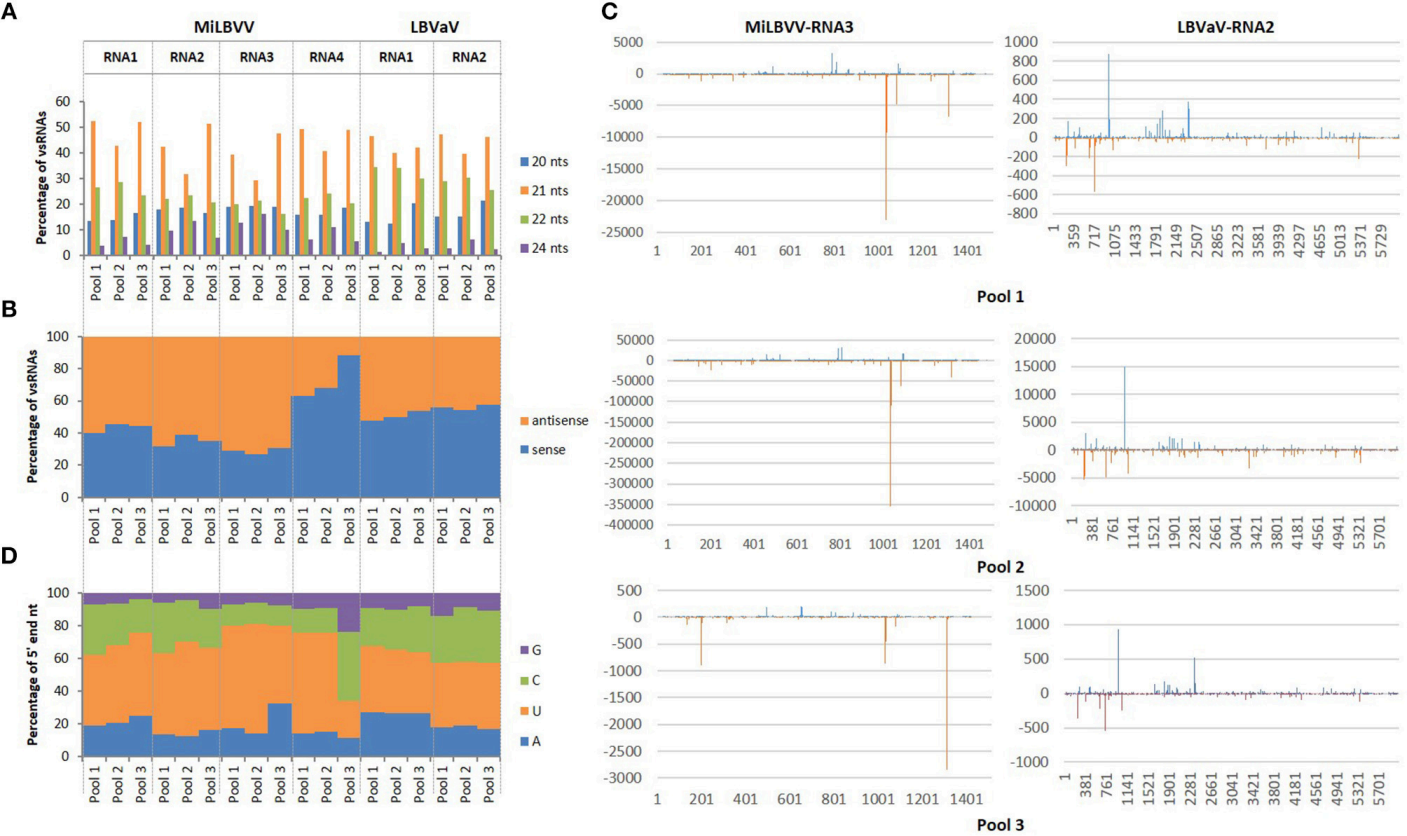
Features of vsRNAs derived from LBVD-associated viruses. **(A)** Size distribution of the more abundant total vsRNAs. **(B)** Orientation distribution of total vsRNAs. **(C)** Distribution of vsRNAs along MiLBVV RNA3 and LBVaV RNA2. **(D)** 5' end nucleotide composition of vsRNAs.

To our knowledge, this was the first time that vsRNAs were sequenced for MiLVBV and LBVaV, so that a bioinformatics pipeline was used to characterize the vsRNA populations in our sRNA-seq datasets. vsRNAs from 20 to 24 nt that perfectly matched the reference viral genomes (isolate LS301-O of MiLBVV and isolate LS302 of LBVaV) encompassed more than 90% of the total vsRNAs for pools 1 and 2, whereas for pool 3 this percentage ranged from 65.5 to 81.2%. The total number of vsRNAs of 20–24 nt was variable among samples and among viral genomes, ranging from 1,654 for MiLBVV-RNA4 in pool 3 to 1,567,448 for MiLBVV-RNA3 in pool 2. Likewise, the number of unique vsRNA sequences varied from 182 for MiLBVV-RNA4 in pool 3 to 12,610 for MiLBVV-RNA1 in pool 2. The size distribution of these vsRNAs was very similar between the three samples analyzed. vsRNAs were mostly 21 nt long (ranging from 29.4 to 52.2%) followed by 22 nt long (ranging from 19.7 to 34.4%) (Figure 3A). vsRNAs derived from RNA1, RNA2, and RNA3 of MiLBVV were mostly of antisense polarity (ranging from 54.6% in RNA2 for pool 2 to 73.1% in RNA3 for pool 2) (Figure 3B). However, vsRNAs derived from RNA4 of MiLBVV were mostly of sense polarity (ranging from 88.4% in pool 3 to 63% in pool 1) (Figure 3B). LBVaV-RNA1-derived vsRNAs were equally of sense and antisense polarity, but LBVaV-RNA2-derived vsRNAs of sense polarity were slightly more abundant (Figure 3B). In agreement with our previous analysis using VirusDetect, vsRNAs were distributed along the complete sequence of the viral genomes (90% of genome coverage in average), although their distribution was heterogeneous and there were vsRNA accumulation peaks located in specific regions of the viral genomes. As an example, we plotted both sense and antisense vsRNAs mapping to the RNA segment encoding the CP gene (RNA3 of MiLBVV and RNA2 of LBVaV) for all three sample pools (Figure 3C). The distribution of accumulation peaks in plots was very similar between samples, although the amount of vsRNAs for both viral genomes was lower for pool 3 than for pool 1 and much lower than for pool 2 (Figure 3C). Lastly, we analyzed the composition of the 5′ end of the vsRNAs, as the loading of sRNAs in the effector complexes is dictated by the identity of this nucleotide (Kim, 2008). Our analysis revealed that most vsRNAs had a 5′ end uridine for all viral genomes and in all three pools analyzed with the only exception of vsRNAs from RNA4 of MiLBVV in pool 3, which mostly had a cytosine in that position (Figure 3D). In general, vsRNAs derived from MiLBVV and LBVaV infecting lettuce shared common characteristics to previously described plant vsRNA populations and also vsRNAs from viruses infecting fungi or animals (Donaire et al., 2009; Parameswaran et al., 2010; Donaire and Ayllón, 2017; Li et al., 2017; Xu and Zhou, 2017; Kaldis et al., 2018; Lan et al., 2018).

### Diversity and Phylogenetic Analysis of the MiLBVV Population

**Figure 4:**
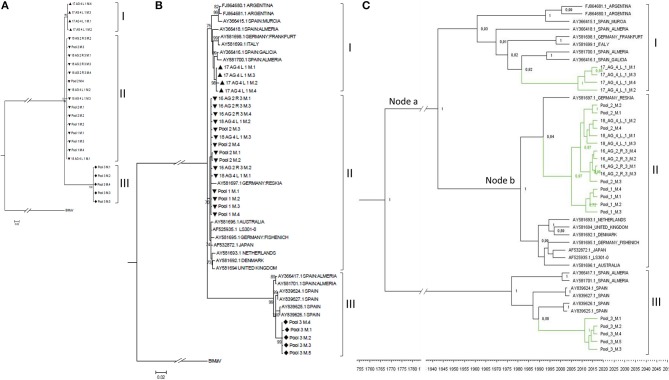
Phylogenetic relationships among the complete CP nucleotide sequences of MiLBVV isolates. The evolutionary history was inferred using the Maximum Likelihood method with 1000 bootstrap replicates, applying the Tamura-Nei+G model. Branch nodes with < 70% bootstrap values were collapsed. Symbols before the sequence names correspond to: Triangles, sequences determined in this work belonging to group I; Upside-down triangles, sequences determined in this work belonging to group II; Diamonds, sequences determined in this work belonging to group III. **(A)** Tree constructed with 24 non-redundant sequences determined in this work and rooted with blueberry mosaic associated virus CP sequence (ref. NC 036634.1). **(B)** Tree constructed including additional MiLBVV sequences from other databases. **(C)** Bayesian Maximum Clade credibility tree. Node labels correspond to posterior probability support values (values below 50% are not shown). The inferred divergence time is shown below the tree. Sequences were named according to the GenBank accession number except for the sequences identified in this study (colored in green), which were named with their sequence codes (Table 1).

The region between nucleotides 12 and 1,325 of MiLBVV RNA3, which encodes the CP, was selected for cloning and subsequent sequencing. We chose to use this strategy instead of direct sequencing of RT-PCR products to avoid uncertainties due to potential mixed infections. A total of 27 cDNA clones were prepared from either individual or pooled samples, and up to 4 cDNA clones were sequenced per RNA extract (Table 1). Sequences were aligned and percentages of nucleotide identity were calculated between pairs of sequences using the Clustal Omega program (Sievers and Higgins, 2014), which ranged between 88.1 and 100%. These percentages ranged from 86.9 to 100% for MiLBVV CP sequences included in the NCBI database (Supplementary Table 3). Non-redundant sequences were aligned and used for phylogenetic analyses (Figure 4), in which we included the blueberry mosaic associated virus (BIMaV, species *Blueberry mosaic associated virus*, genus *Ophiovirus*) CP sequence (NC 036634.1) as an outgroup. A first analysis in which only the sequences determined in this study were considered showed the existence of three branches in the phylogenetic tree (Figure 4A). The sequences within each branch were highly similar (99.3–99.9% nucleotide identity), while pairs of sequences from different branches were more dissimilar (87.7–97.8% nucleotide identity). This phylogenetic analysis was complemented with a population analysis based on genetic distances, which allow for the estimation of the degree of genetic variation within and between populations (Nei et al., 2001). Distances among pairs of sequences were estimated using the Tamura-Nei method (Tamura, 1992; Tamura and Nei, 1993). Results showed that the mean nucleotide distance among the whole set of sequences was 0.052 ± 0.004, whereas for sequences within branches these values ranged from 0.004 ± 0.001 to 0.001 ± 0.000, suggesting genetic differentiation. Therefore, our analyses suggested the existence of at least three viral strains in the geographic area under study.

MiLBVV nucleotide sequences from the NCBI database (Supplementary Table 3) were included in a second phylogenetic analysis. The basic branching pattern for this tree was similar to the previous one, again identifying three main lineages (Figure 4B). The percentages of nucleotide identity among pairs of sequences within the three main branches ranged between 96.1 and 99.9% for branch I, 97.9 to 99.9% for branch II, and 97.6 and 99.9% for branch III, supporting the existence of these three well differentiated lineages. While branch III grouped only Spanish sequences, for branches I and II there was no apparent relationship between geography (place where the isolate was from) and position in the tree. Thus, the Spanish sequences of branch I appeared related to other earlier Spanish sequences from Almería, Murcia and Galicia, but also to one sequence from Italy, one from Germany and two from Argentina, and the Spanish sequences in branch II appeared related to one from Germany (Figure 4B). Phylogenetic reconstruction using Bayesian methods suggested that diversification of groups I and II occurred ~76.2 years ago (Node a; HPD 95% 24.6–384 years), and that the lineage including the most prevalent type of sequences in this study diverged from a German sequence ~28.2 years ago (Node b; HPD 95% 14.8–53.8 years) (Figure 4C).

An analysis of the direction and intensity of the selection acting on the CP was also carried out (Table 4). The number of synonymous (dS) and non-synonymous (dN) substitutions between pairs of sequences was estimated to determine the coefficient dN/dS, which when >1 suggests that the gene is under positive selection, when < 1 suggests that the gene is under negative or purifying selection, and when equal to 1, the gene is under neutral selection. In general, the dN/dS ratios estimated for the nucleotide sequences were smaller than 1 (Table 4), suggesting that the MiLBVV CP gene was under negative, purifying selection.

**Table 4:** Average number of nucleotide substitutions^a^ among MiLBVV and LBVaV CP coding regions.

**Virus**	**dN^b^**	**dS^c^**	**dN/dS**
MiLBVV	0.013 ± 0.002	0.224 ± 0.021	0.058
MiLBVV Clade I	0.009 ± 0.002	0.053 ± 0.007	0.169
MiLBVV Clade II	0.004 ± 0.001	0.019 ± 0.004	0.211
MiLBVV Clade III	0.002 ± 0.001	0.036 ± 0.007	0.056
MiLBVV from Águilas	0.011 ± 0.002	0.218 ± 0.022	0.050
LBVaV	0.005 ± 0.001	0.043 ± 0.006	0.116
LBVaV Clade I	0.005 ± 0.001	0.031 ± 0.005	0.161
LBVaV Sub-Clade Ia	0.005 ± 0.001	0.026 ± 0.004	0.192
LBVaV Sub-Clade Ib	0.005 ± 0.002	0.023 ± 0.006	0.217
LBVaV Clade II	0.001 ± 0.001	0.021 ± 0.006	0.048
LBVaV from Águilas	0.002 ± 0.000	0.014 ± 0.004	0.143

a *Estimated using the Nei and Gojobori (Nei and Gojobori, 1986) or Kimura 2-parameter (Kimura, 1980) methods for MiLBVV or LBVaV, repectively. Sequences are those included in phylogenetic analysis in Figures 4B, 5A; values have been estimated also for the Spanish sequences determined in this work (“from Águilas”)*.

b *Mean nucleotide diversity in non-synonymous positions*.

c *Mean nucleotide diversity in synonymous positions*.

### Diversity and Phylogenetic Analysis of the LBVaV Population

As for MiLBVV, cDNAs for the LBVaV CP gene were cloned and sequenced, generating 29 good quality sequences from cDNA clones derived from either individual or pooled samples. Up to 4 cDNA clones were sequenced per RNA extract (Table 1). Sequences were aligned and nucleotide identities were calculated; nucleotide identities varied from 98.8 to 100%, already showing lesser diversification of the LBVaV population compared to the MiLBVV population. When LBVaV CP sequences from the NCBI database (Supplementary Table 2) were included in the analysis, percentages of nucleotide identity varied from 93.9 to 100%. Phylogenetic analyses (Figure 5) were carried out including the 23 non-redundant sequences determined here. The sequence of the tobacco stunt virus (TStV; species *Tobacco stunt virus*, genus *Varicosavirus*) CP gene (ref AB190525.1) was used as the outgroup. In this case, due to the low variability in the population, an analysis with only the sequences determined in this study resulted in trees with low confidence branching patterns, independently of the phylogenetic reconstruction method used (data not shown). In contrast, when nucleotide sequences from the NCBI database (Supplementary Table 2) were included, phylogenies were fully informative. Two main nodes with bootstrap values above 95% could be identified; the branches radiating from these nodes grouped sequences from common geographical origins: Branch I grouped European and Australian sequences and branch II grouped Japanese sequences (Figure 5A). Branch I could be differentiated into linages Ia that grouped European sequences and lineage Ib, which included Australian sequences. The percentages of nucleotide identity for sequences within branches I, Ia, Ib and II ranged between 95.5–99.9%, 95.5–99.9%, 98.3–99.7%, and 99.1–99.75%, respectively, whereas for sequences between branches, the percentages ranged between 95.5–98.4%, 93.9–96.9%, and 95.73–96.9% for the pairs Ia-Ib, Ia-II, and Ib-II, respectively.

**Figure 5:**
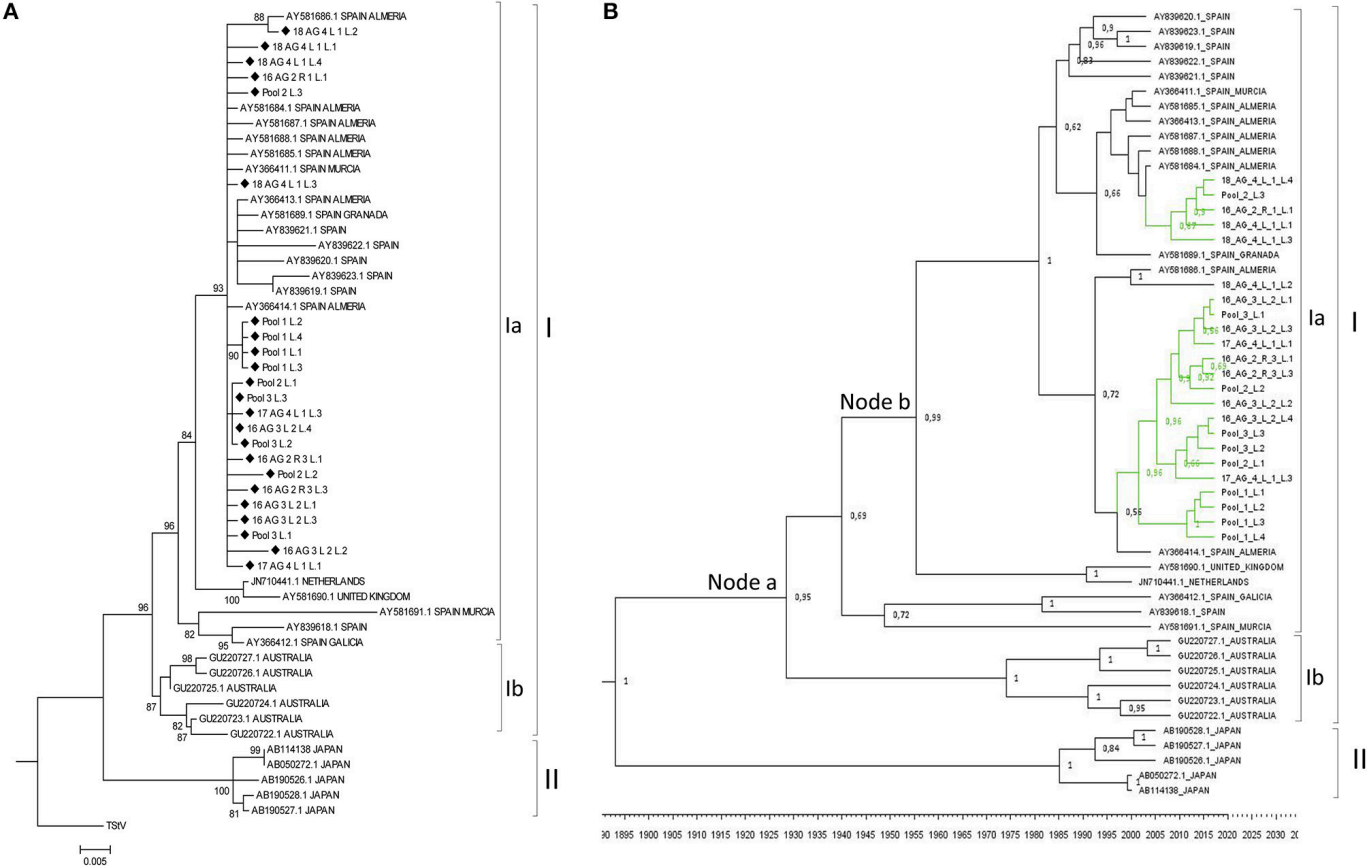
**(A)** Phylogenetic relationships among the complete CP nucleotide sequences of LBVaV isolates. The evolutionary history was inferred using the Maximum Likelihood method with 1000 bootstrap replicates, applying the Kimura-2-parameter+G model. Branch nodes with < 70% bootstrap values were collapsed. Tree including the 25 non-redundant sequences determined in this work and LBVaV sequences from Australia, Europe and Japan. The tree was rooted with tobacco stunt virus (TStV; genus *Varicosavirus*) CP sequence (ref AB190525.1). Sequences determined in this work are marked with diamond symbols. **(B)** Bayesian Maximum Clade credibility tree. Node labels correspond to posterior probability support values (values below 50% are not shown). The inferred divergence time is shown below the tree. Sequences were named according to the GenBank accession number except the sequences identified in this study (colored in green), which were named with their sequence codes (Table 1).

A population analysis based on genetic distances (Nei et al., 2001), in this case estimated using the Kimura 2-parameter method (Kimura, 1980), showed that the mean nucleotide distance among the LVBaV sequences determined in this work was 0.005 ± 0.001, again illustrating the lesser diversity found in the LVBaV population compared to MiLBVV. For the whole set of sequences used in the phylogenetic analysis, this datum was 0.018 ± 0.002. Within branches Ia, Ib and II, the mean nucleotide distances were 0.010 ± 0.001, 0.011 ± 0.002, and 0.007 ± 0.002, respectively, whereas these values for sequences between branches were 0.025 ± 0.005, 0.046 ± 0.006, and 0.040 ± 0.004 for the pairs Ia-Ib, Ia-II, and Ib-II, respectively. These data, together with our phylogenetic analyses, support the existence of LVBaV populations differentiated according to geography. Bayesian analysis suggested that the European and Australian sequences diverged 91.55 years ago (Node a; HPD 95% 48.4–157.3 years), and the Spanish sequences diverged from sequences from the United Kingdom and The Netherlands 62.8 years ago (Node b; HPD 95% 35.4–103 years) (Figure 5B); interestingly, the latter sequences shared ancestors with again Spanish sequences, suggesting the exchange of LBVaV isolates within the European territory. As before, dS and dN substitutions between pairs of sequences were estimated to determine the dN/dS ratios (Table 4). Independently of the subpopulation considered, these ratios were always below 1, which suggests that the LBVaV CP gene was also under negative selection.

### MiLBVV and LBVaV Detection in Additional Field Samples, Including Seeds

**Figure 6:**
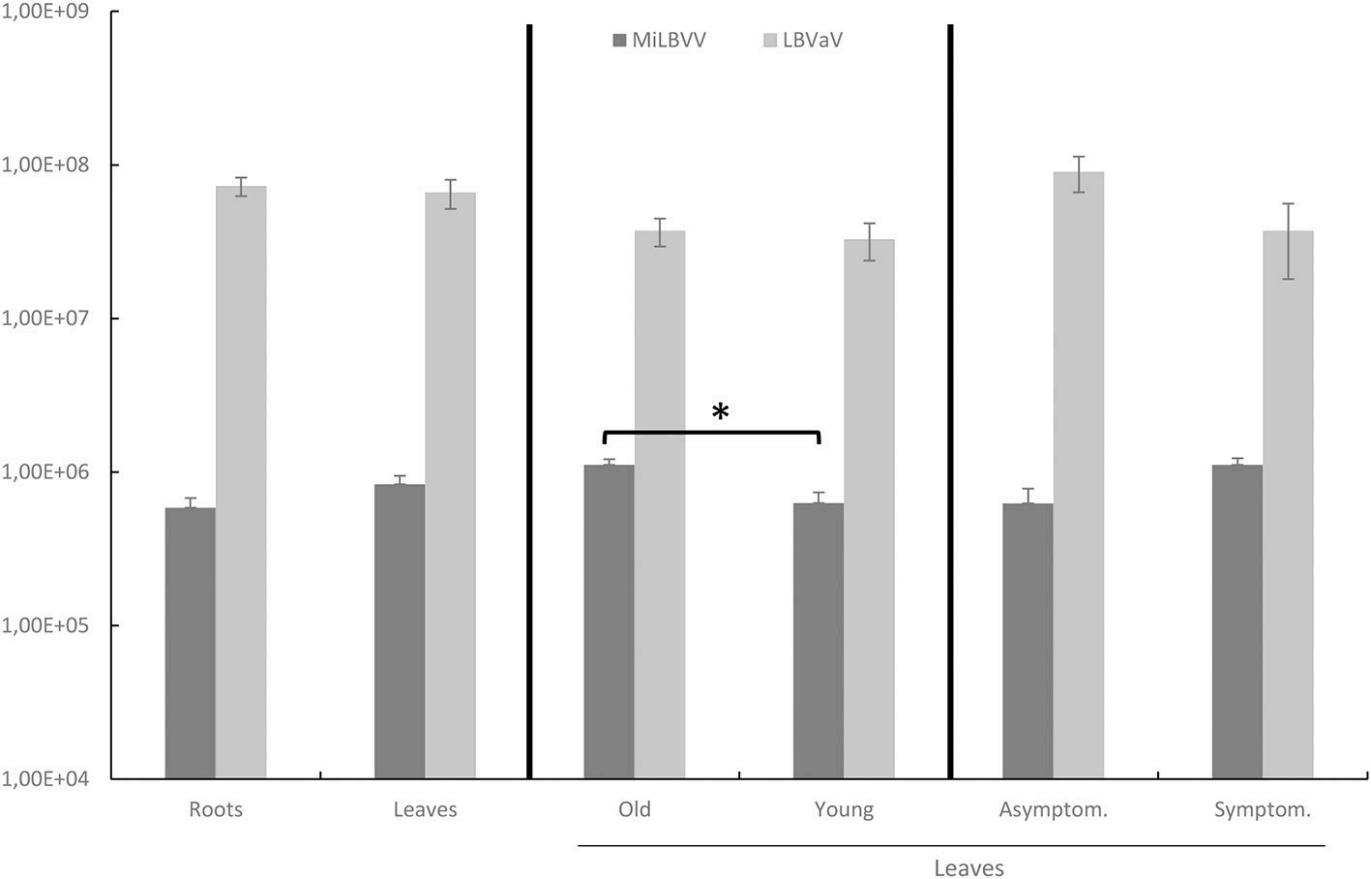
Accumulation of viral CP RNA in tissues from different lettuce samples from affected fields. Statistical analyses were performed separately comparing the accumulation of both viruses in leaves vs. roots, old leaves vs. young leaves, and asymptomatic vs. symptomatic leaves. Data represent the mean ± SE of each group. An asterisk indicates significant differences according to a Kruskal-Wallis's test (*p* < 0.05).

An alignment of LBVaV CP consensus sequences from the sRNA-seq analysis above and LBVaV sequences available in the NCBI database allowed for the design of conserved primers for this virus. We also checked sequence conservation for the MiLBVV primers designed by Momonoi et al. (2015) using the sequences determined in this study. The complete set of primers (Table 2) can amplify fragments in the CP genes of both viruses. Using these primers, we analyzed the presence of the two viruses in new lettuce samples to (i) detect the presence of both viruses in samples from commercial crops and seed lots and (ii) identify the best tissues to sample for virus detection. We thus sampled leaves and roots of 26 lettuce plants from fields from Águilas (Murcia, Spain) which were very affected by LBVD; out of these, 14 plants were asymptomatic. All leaf samples were positive for LBVaV, as were roots, but differences were observed for MiLBVV; all symptomatic and 85.7% of the asymptomatic leaf samples were infected by MiLBVV, whereas 58.3 and 92.9% of the root samples from symptomatic and asymptomatic plants, respectively, were infected by MiLBVV. We then hypothesized that qualitative MiLBVV detection discrepancies in roots and leaves could be due to differences in virus accumulation in the different tissues. Therefore, we next studied the accumulation of both viruses in leaves vs. roots, young leaves vs. old leaves, and symptomatic vs. asymptomatic leaves (Figure 6). Our data showed that LBVaV accumulated to higher levels than MiLBVV, and differences for the different classes of tissues (leaves, roots, symptomatic, asymptomatic) were only statistically significant for old vs. young MiLBVV-infected leaves (Figure 6). This is notorious in the case of asymptomatic plants, which accumulated equivalent amounts of viral RNA than symptomatic plants. We also analyzed seeds from 11 different varieties from 5 different commercial providers (Table 5). For each virus we used two PCR-based methods, particularly a confirmatory method using TaqMan probes (Table 2) to improve specificity. Both methods provided similar results, seeds from varieties 1 and 2 were positive for both viruses, seeds from varieties 3, 4, 5, 6, and 9 were positive by LBVaV only, and seeds from varieties 7, 8, 10, and 11 were negative (Table 5). To confirm these results and for further analysis, the regions encoding viral CPs were cloned and sequenced. Two cDNA clones were obtained for MiLBVV and 15 for LBVaV from positive seeds samples (Table 1). Phylogenetic analyses including these sequences showed that MiLBVV seed sequences grouped with lineage II sequences, which included most of the sequences determined in this work for MiLBVV (Supplementary Figure 1). The 15 LBVaV seed sequences were grouped in lineage Ia, which included the European and Spanish sequences analyzed in this work (Supplementary Figure 2).

**Table 5:** Results of qRT-PCR assays for detection of the viruses associated with LBVD in commercial lettuce seeds obtained from different providers.

**Samples^a^**	**MiLBVV**		**LBVaV**	
	**SYBR Green**	**TaqMan**	**SYBR Green**	**TaqMan**
17_AG_Ø_S1_Ø	**+**	**+**	**+**	**+**
17_AG_Ø_S2_Ø	**+**	**+**	**+**	**+**
17_AG_Ø_S3_Ø	**–**	**–**	**+**	**+**
17_AG_Ø_S4_Ø	**–**	**–**	**+**	**+**
17_AG_Ø_S5_Ø	**–**	**–**	**+**	**+**
17_AG_Ø_S6_Ø	**–**	**–**	**+**	**+**
17_AG_Ø_S7_Ø	**–**	**–**	**–**	**–**
17_AG_Ø_S8_Ø	**–**	**–**	**–**	**–**
17_AG_Ø_S9_Ø	**–**	**–**	**+**	**+**
17_AG_Ø_S10_Ø	**–**	**–**	**–**	**–**
17_AG_Ø_S11_Ø	**–**	**–**	**–**	**–**

a *Each sample corresponds to a different variety of lettuce (1–11) and was named according to the following code: Year_Location_Plot_Tissue_Plant. ∅ indicates absence of data*.

## Discussion

Our results have confirmed (see for example Zaagueri et al., 2017) that VirusDetect coupled to sRNA-seq is an efficient tool for identifying viruses infecting plants from commercial crops. In agreement with previous work, the viruses detected in association with LBVD were MiLBVV and LBVaV, although we also identified sequence reads corresponding to RWMV and MNSV in pools 2 and 3, respectively. The relevance of RWMV remains to be determined, as we were unable to confirm its presence using qRT-PCR (data not shown) in spite of genome coverage in pool 2 equivalent or slightly smaller than that for MiLBVV or LBVaV and the use of two different primer pairs in RT-PCR. In this regard, a recent study comparing the sensitivity of sRNA-seq vs. RT-PCR for virus identification using total RNA from different species showed that their detection limits were very similar; however, sRNA-seq was 10 times more sensitive than RT-PCR for detection of already-known viral genomes (Santala et al., 2018). In the case of MNSV, its detection was surely negligible and probably associated to the sampling of lettuce roots, perhaps contaminated with sporangia of viruliferous *Olpidium bornovanus*, the MNSV vector, from cucurbit crops that rotated in the area before lettuce. VirusDetect also has the potential for finding mixed strain infections; we identified different contigs in one of our sample pools with variable similarity to the reference MiLBVV genome, showing that the identification of contigs belonging to different virus strains is feasible using this suite of programs. However, the VirusDetect potential for identification of the closest isolate present in a sequenced sample among all sequences in databases is limited. The use of a broader plant virus database containing all possible sequences could overcome this limitation, although it may not be feasible in practical terms due to excessive computing resources needs. Here, by using a local BLASTN alignment of the viral contigs against different CP sequences of MiLBVV and LBVaV, we found that the viral contigs in our samples were closely related to MiLBVV isolates from the Netherlands and Spain and LBVaV isolates from Spain. The subsequent Sanger sequencing of the CP sequences of the sRNA-seq sample pools and the phylogenetic analyses confirmed these results.

Using the sRNA-seq data, we have described for the first time the vsRNA populations from MiLBVV and LBVaV. vsRNAs play an essential role on the antiviral RNA silencing defense mechanisms in plants and may also participate in the regulation of host gene expression during viral infection (Llave, 2010). We showed that the characteristics of these vsRNA populations were essentially similar to those described for other plant viruses in terms of size, orientation and 5′ end nucleotide composition. These results suggest that the different sRNA biogenesis pathways are fully operative in lettuce and are probably the same than in other plant species, as suggested by the fact that most endogenous sRNAs corresponded to 24-nt heterochromatic small interfering RNAs and because conserved microRNAs were previously identified in lettuce (Reyes-Chin-Wo et al., 2017). The vsRNA profiles from our three different sample pools were almost identical, although some differences were found. For instance, the number of vsRNAs derived from all viral genomes in pool 2 was much higher than in pools 1 and 3, which could be correlated with higher viral replication or accumulation in samples in this pool, as was confirmed by qRT-PCR amplification (Supplementary Figure 3). Moreover, the higher percentage of coverage found for all viral genomes in pool 2, as compared with the other sample pools (Supplementary Table 1) could be a reflection of high viral accumulation, as it has been described that high viral amounts result in an almost complete coverage of vsRNAs along the reference genomes (Santala et al., 2018). vsRNAs derived from MiLBVV and LBVaV in the three sample pools were mostly 21 and 22 nts in length, which suggests that the same dicer-like ribonucleases, likely to be DCL4 and DCL2, are involved in their biosynthesis, as for vsRNAs derived from any viral genome characterized to date (Donaire et al., 2008). With the exception of the MiLBVV RNA4, MiLBVV-derived vsRNAs of antisense polarity were predominant in the data sets. In the case of LBVaV, vsRNAs of sense and antisense polarity accumulated in a similar proportion or with a small bias toward vsRNAs of sense polarity in RNA2. For both viruses, the negative strands carry the genetic information, thus, it is possible that the accumulation of the negative-strands and/or their half-lives during viral infection cycles are higher than those of the positive strands, explaining results for MiLBVV RNAs 1, 2, and 3. Similar amounts of vsRNAs of both polarities could be explained if vsRNAs proceeded from viral dsRNAs formed during the replication cycle or by the activity of plant RNA dependent RNA polymerases (RDRs), as described for a negative-stranded RNA mycovirus (Donaire and Ayllón, 2017). The remarkable amount of vsRNAs of sense polarity derived from MiLBVV-RNA4 in all sample pools could be explained if the positive strand was able to form more abundant or perhaps different stable secondary structures than the negative strand due to thermodynamic reasons. vsRNAs showed a near full-coverage of the viral genomes for both MiLBVV and LBVaV (Supplementary Table 1 and Figure 2), however, the distribution of vsRNAs along the viral genomes was heterogeneous, showing peaks of vsRNA accumulation in certain regions and in both viral strands (Figure 3). Heterogeneous vsRNA distribution has been reported roughly in all plant viruses studied to date, and could be a reflection of regions that are preferentially targeted by DCL for vsRNA biogenesis, such as highly structured regions with strong secondary structure, although clear experimental evidence for this is still lacking (Donaire et al., 2009; Llave, 2010; Donaire and Ayllón, 2017).

Knowledge on the variability and genetic structure of viral populations is fundamental for the deployment of sustainable disease control strategies (García-Arenal et al., 2001). Our analyses of the MiLBVV populations indicated that isolates of three different lineages co-circulated during the epidemics in Spain. The diversity of the sequences within each lineage was very low, while the total diversity was high. This denotes that the genetic diversity of the analyzed population was determined by the presence of these three well-differentiated lineages and not by the diversity of isolates within the lineages. On the other hand, the overall diversity of the sequences determined in this work (mean nucleotide distance = 0.059 ± 0.005) was almost as great as that determined for the set of these sequences plus those that could be downloaded from the databases (0.068 ± 0.005), which indicates that the isolates of the Spanish population mirror almost all of the diversity described for the MiLBVV species (Maccarone et al., 2010b). Likewise, in the phylogenetic analysis in which non-Spanish isolates were included, no relationship was found between the geographic origin of the sequence and its position in the phylogenetic tree, which may suggest frequent long distance movement of infective materials. The situation described for LBVaV was different. On the one hand, the diversity of the population analyzed was considerably lower than that of the MiLBVV population; essentially, the population of LBVaV analyzed in this work could be considered genetically undifferentiated. In contrast, when a phylogenetic analysis was carried out including the sequences determined in this work plus others from diverse geographical origins available in databases, the existence of three main lineages that shared common geographic origins (Japan, Australia, and Europe) could be identified, in agreement with previous observations (Maccarone et al., 2010c). Thus, for LBVaV there seems to be little diversity at the regional scale, although there is differentiation at the global scale. These observations argue in favor of a limited movement of LBVaV infective material at the global scale, contrary to what was discussed for MiLBVV. Lastly, the greater genetic diversification of MiLBVV in the study area compared to LBVaV may suggest a presence of the first virus in the zone prior to the second one, although the Bayesian analyses that we have carried out seemed to suggest otherwise.

The primers used to detect and quantify the accumulation of MiLBVV and LBVaV in this work functioned effectively, providing conclusive results from all types of samples. With regard to the analysis of symptomatic and asymptomatic plants, our results coincided with those of other authors (Walsh, 1994; Araya et al., 2011) in the sense that asymptomatic plants could accumulate similar amounts of viruses as symptomatic plants, and that the severity of symptoms was not associated with a greater accumulation of viruses. This could have a very significant impact when eradicating infection foci and/or virus reservoirs, which would not be perceived by the farmer unless the symptoms were manifested or specific detection of the viruses was carried out with methods such as those used in this work. Thus far, the factors responsible for the expression of the LBVD symptoms are not well known, although the perception of symptoms in the fields is much more frequent in cool periods and short days. Likewise, our results on the accumulation of viruses in infected plants essentially coincided with those of Navarro et al. (2004), although these authors described a greater accumulation of both viruses in old vs. young leaves and roots that our results were not able to reproduce; perhaps this difference was due to the different methods used for detection, in our case more sensitive and probably more appropriate for the quantification of viral accumulation. Another aspect coinciding with the work of Navarro et al. (2004) consisted of the detection of consistently higher accumulation levels for LVBaV than for MiLBVV; this aspect may have had an impact on the characterization of the etiology of the disease (Roggero et al., 2003; Sasaya et al., 2008) as the detection of MiLBVV requires a higher sensitivity than that required for LBVaV. Also, the distribution of viruses along with the sampling of infected plants may have played an important role in this regard. In summary, our work points to a consistent association of both viruses with LBVD in Murcia fields. On the other hand, our results of virus detection in seeds suggested the possibility of their transmission through seeds. The literature regarding the presence of both viruses in seeds is very scarce. Maccarone (2009) detected the presence of LBVaV and MiLBVV by RT-PCR in extracts of seeds from lettuce plants affected by LBVD. In our work, the presence of both viruses was detected in commercial seed lots from different brands. In all the batches analyzed, virus titer, both for MiLBVV and for LBVaV, was very low, so if there was seed transmission, the number of infected seedlings would probably be very low. LBVaV was detected more frequently than MiLBVV in the analyzed seeds, which would suggest a higher probability of seed transmission for LBVaV than for MiLBVV, although our phylogenetic analyses suggest otherwise. In any case, the detection of both viruses in commercial seed lots is an important finding and precautionary measures should be adopted, in addition to carrying out further studies to determine if the presence of the viruses in the seeds can result in the infection of the resulting seedlings.

## Author Contributions

MA, MJ, MS-P, YH, and AB-V designed and planned the research. AB-V performed the experiments, carried out statistical analyses and was involved in data interpretation and manuscript writing with MA and LD. LD was involved in the bioinformatic analyses of viral small RNA. CT and CG-A were involved in the diversity and phylogenetic analyses. YH coordinated and managed research activities.

### Conflict of Interest Statement

The authors declare that the research was conducted in the absence of any commercial or financial relationships that could be construed as a potential conflict of interest.

## Footnotes

^1^http://www.fao.org/faostat/es/#data/TP

^2^https://www.mapama.gob.es/es/estadistica/temas/estadisticas-agrarias/agricultura/esyrce/

^3^http://www.bioinformatics.babraham.ac.uk/projects/fastqc/

^4^https://github.com/ldonaire/Perl-scripts

## Supplementary Material

Supplementary Figure 1 Phylogenetic relationships among the complete CP nucleotide sequences of MiLBVV isolates, including seed sequences (marked in red). The evolutionary history was inferred by Maximum Likelihood with 1000 bootstrap replicates, with the application of the Tamura-Nei+G model. Branch nodes with < 70% bootstrap values were collapsed. **(A)** Tree constructed with the 26 non-sequences obtained in this work and rooted with blueberry mosaic associated virus (ref. NC 036634.1) CP sequence. **(B)** Tree constructed including additional MiLBVV from the databases. **(C)** Bayesian Maximum Clade credibility tree. Node labels correspond to posterior probability support values (values below 50% are not shown). The inferred divergence time is shown below the tree. Sequences were named according to the GenBank accession number except for the sequences identified in this study (colored in green), which were named with their sequence codes (Table 1).

Supplementary Figure 2 Phylogenetic relationships among the complete CP nucleotide sequences of LBVaV isolates, including sequences from seeds (marked in red). The evolutionary history was inferred using the Maximum Likelihood method with 1000 bootstrap replicates, with the application of the Kimura-2-parameter+G model. Branch nodes with < 70% bootstrap values were collapsed. **(A)** Tree including the 38 non-redundant sequences determined in this work and LBVaV sequences from Australia, Europe and Japan. It was rooted with tobacco stunt virus (TStV; genus *Varicosavirus*) CP gene (ref AB190525.1). **(B)** Bayesian Maximum Clade credibility tree. Node labels correspond to posterior probability support values (values below 50% are not shown). The inferred divergence time is shown below the tree. Sequences were named according to the GenBank accession number except for the sequences identified in this study (colored in green), which were named with their sequence codes (Table 1).

Supplementary Figure 3 Accumulation of viral CP RNA in pools 1–3 (see Table 1). Data represent the mean ± SE for each pool.

Supplementary Table 1 Coverage and sequencing depth of sRNAs mapping to viruses identified by the VirusDetect online tool.

Supplementary Table 2 Percentage of identity of viral contigs assembled by VirusDetect against CP sequences of LBVD-associated viruses collected from NCBI.

Supplementary Table 3 List of MiLBVV and LBVaV sequences from NCBI used for the phylogenetic analysis.
