# Supplementary figures and images for: Small RNA-Seq to Characterize Viruses Responsible of Lettuce Big Vein Disease in Spain
Source: Front Microbiol. 2018 Dec 21;9:3188. doi: 10.3389/fmicb.2018.03188 (PMC6309106; doi:10.3389/fmicb.2018.03188)

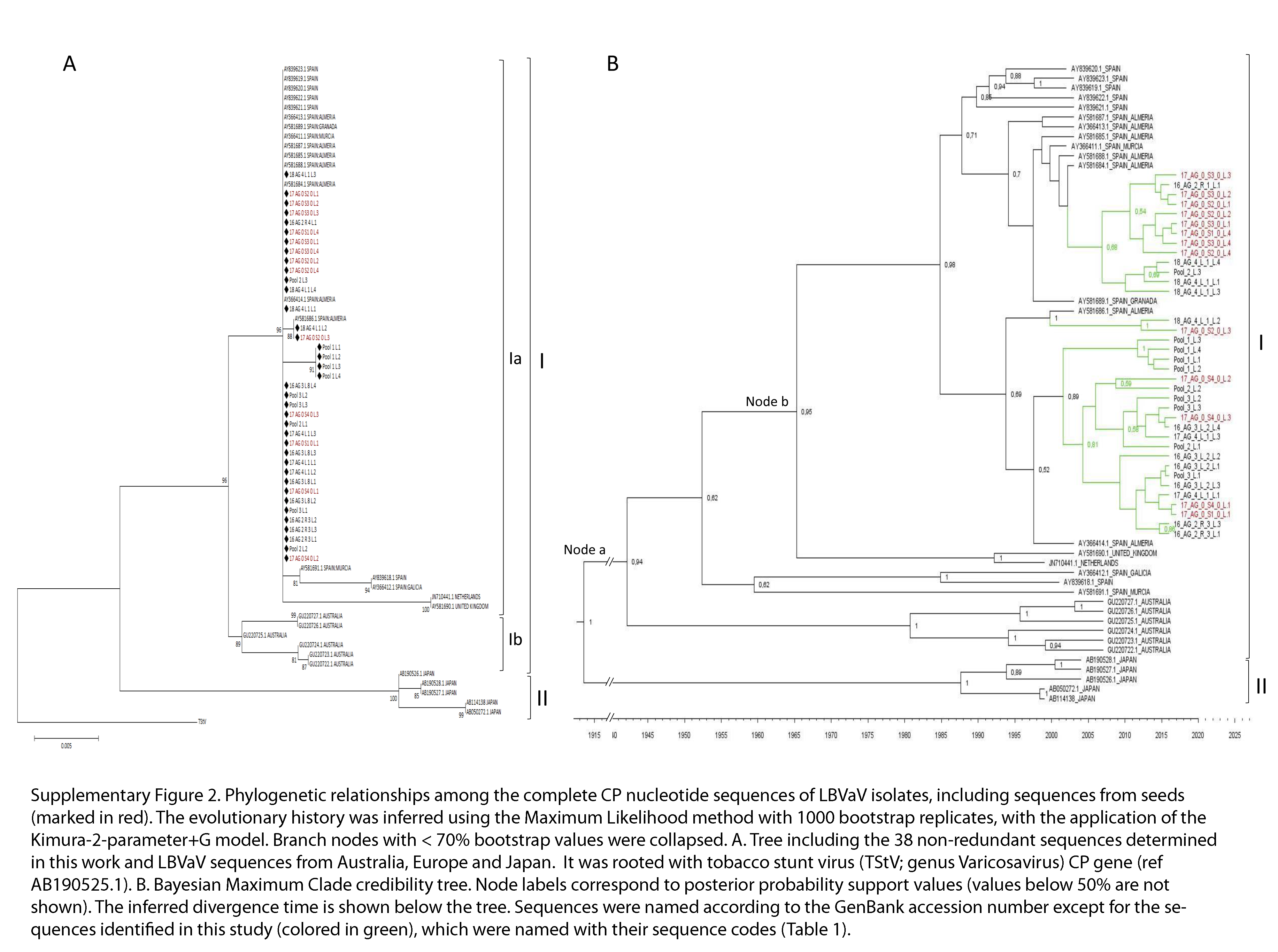

Supplement: Supplementary Figure 2 — Phylogenetic relationships among the complete CP nucleotide sequences of LBVaV isolates, including sequences from seeds (marked in red). The evolutionary history was inferred using the Maximum Likelihood method with 1000 bootstrap replicates, with the application of the Kimura-2-parameter+G model. Branch nodes with < 70% bootstrap values were collapsed. (A) Tree including the 38 non-redundant sequences determined in this work and LBVaV sequences from Australia, Europe and Japan. It was rooted with tobacco stunt virus (TStV; genus Varicosavirus) CP gene (ref AB190525.1). (B) Bayesian Maximum Clade credibility tree. Node labels correspond to posterior probability support values (values below 50% are not shown). The inferred divergence time is shown below the tree. Sequences were named according to the GenBank accession number except for the sequences identified in this study (colored in green), which were named with their sequence codes (Table 1). [file Image_2.tif]

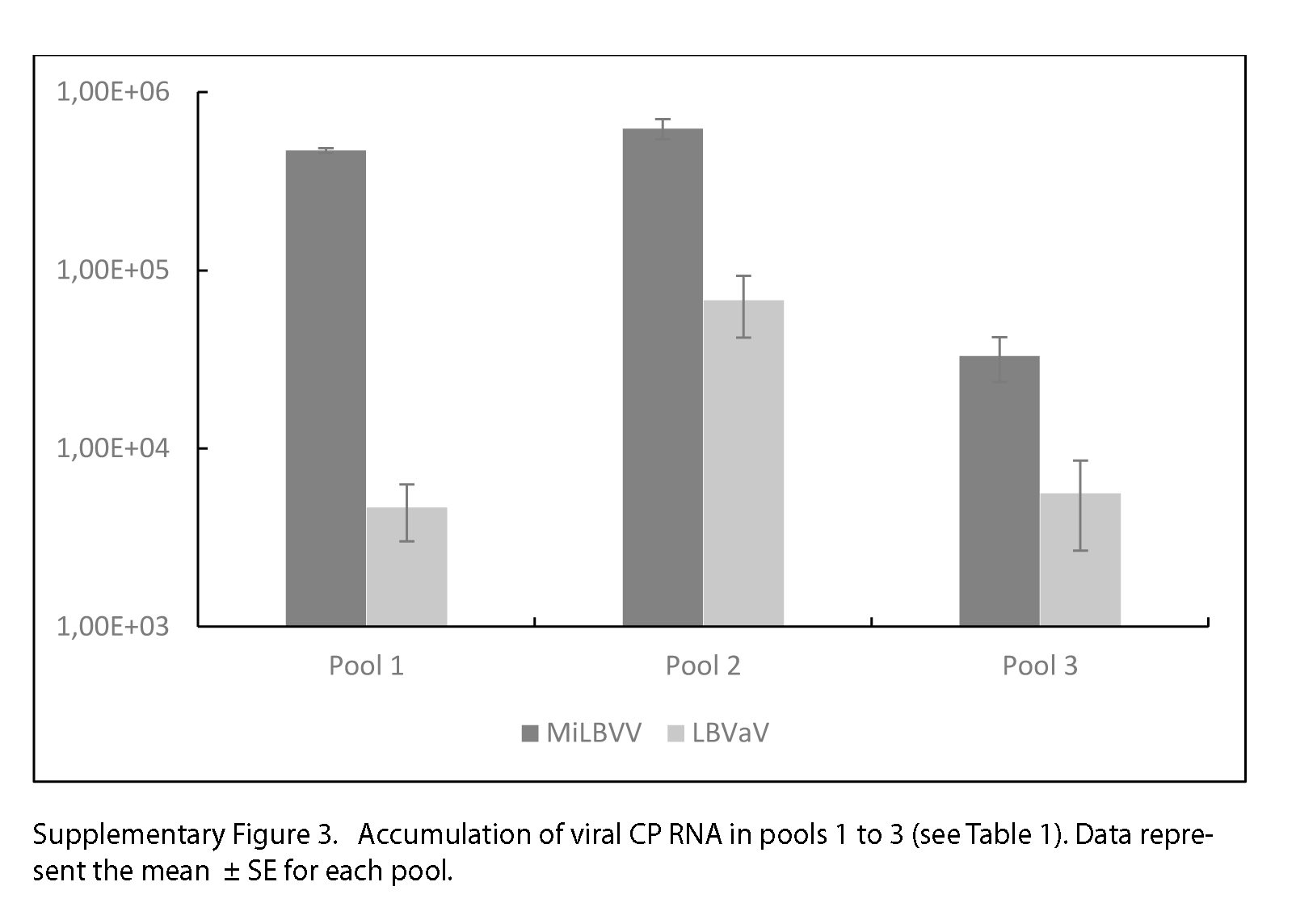

Supplement: Supplementary Figure 3 — Accumulation of viral CP RNA in pools 1–3 (see Table 1). Data represent the mean ± SE for each pool. [file Image_3.tif]
